# Changes in the pH value of the human brain in Alzheimer’s disease pathology correlated with CD68-positive microglia: a community-based autopsy study in Beijing, China

**DOI:** 10.1186/s13041-025-01180-3

**Published:** 2025-02-10

**Authors:** Xue Wang, Xiangqi Shao, Liang Yu, Jianru Sun, Xiang-Sha Yin, Zhen Chen, Yuanyuan Xu, Naili Wang, Di Zhang, Wenying Qiu, Fan Liu, Chao Ma

**Affiliations:** 1 National Human Brain Bank for Development and Function, Institute of Basic Medical Sciences Chinese Academy of Medical Sciences, School of Basic Medicine Peking Union Medical College, Beijing, 100005 China; 2 Department of Human Anatomy, Histology and Embryology, Neuroscience Center, Institute of Basic Medical Sciences Chinese Academy of Medical Sciences, School of Basic Medicine Peking Union Medical College, Beijing, 100005 China; 3 Experimental Teaching Center, School of Basic Medicine, Institute of Basic Medical Sciences Chinese Academy of Medical Sciences, School of Basic Medicine Peking Union Medical College, Beijing, 100005 China; 4 State Key Laboratory of Common Mechanism Research for Major Diseases, Institute of Basic Medical Sciences, Chinese Academy of Medical Sciences and Peking Union Medical College, Beijing, 100005 China; 5 Chinese Institute for Brain Research, Beijing, 102206 China

**Keywords:** Alzheimer’s Disease, Human Brain Bank, Microglia, pH value, Postmortem brain

## Abstract

The microenvironment of the central nervous system is highly complex and plays a crucial role in maintaining the function of neurons, which influences Alzheimer’s disease (AD) progression. The pH value of the brain is a critical aspect of the brain microenvironment in regulating various physiological processes. However, the specific mechanisms and role of this mechanism are not yet fully understood. To better understand the relationship between brain pH and AD, we analyzed the brain pH of the frontal lobe and AD pathology scores in postmortem brain samples from 368 donors from the National Human Brain Bank for Development and Function, 96 of whom were diagnosed with AD pathology. Analysis revealed a significant decrease in brain pH in AD patients, which was strongly correlated with β-amyloid plaques and phosphorylated tau proteins. Here, we elucidated the differential protein expression level of CD68-positive microglia between control and AD groups (t = 3.198, df = 20, *P* = 0.0045), and its protein expression level was correlated negatively with the brain pH value (F = 26.93, *p* = 0.0006). Our findings revealed that increased activation of CD68-positive microglia and disrupted lysosomal homeostasis in the pathological brain tissue of individuals with AD may lead to a decrease in brain pH.

**Supplementary Information:**

The online version contains supplementary material available at 10.1186/s13041-025-01180-3.

## Introduction

Alzheimer’s disease (AD) is a progressive neurodegenerative disorder with the highest incidence characterized by the accumulation of amyloid β-protein (Aβ) plaques, neurofibrillary tangles (NFTs), neuroinflammation, and loss of neurons in the brain [1–5]. It is the most common cause of dementia and affects millions of individuals worldwide, placing a significant economic burden on society and families [6, 7]. Despite extensive research, the exact mechanisms underlying the pathogenesis of AD remain elusive. The pH of the brain may play a crucial role in the occurrence and progression of AD. Protein folding and enzyme activity are highly sensitive to changes in pH, with low pH values contributing to protein aggregation, such as β amyloid peptides [8–10]. Accumulating evidence suggests that disruptions in brain pH value homeostasis may significantly contribute to the pathogenesis and progression of AD and that the maintenance of brain pH values is critical for ensuring optimal neuronal function and survival [11–15]. Alterations in brain pH value regulation have been observed in AD, resulting in an acidic microenvironment that impairs cellular function [12, 16, 17]. The maintenance of brain pH value homeostasis is a multifaceted process influenced by several factors, including neuroinflammation, the accumulation of Aβ peptides, and the presence of cancerous cells [13, 18, 19].

The microenvironment of the brain is strictly regulated by the blood-brain barrier, a physiological barrier separating peripheral blood circulation from the brain parenchyma [20–23]. Disruption of the blood-brain barrier is a key feature of neuroinflammation, which can both originate from and affect the blood-brain barrier [24–26]. However, the exact connection between these processes and changes in the pH of brain tissue is still unclear. This research aims to explore the associations between decreased pH in AD brain tissues and pathological changes, as well as the roles of microglia in this process.

In the present study, we analyzed the pH values of brain donor samples from patients with neurodegenerative pathologies and normal brain donor samples from patients without brain neurodegenerative disorders. Furthermore, we conducted studies using immunohistochemistry to investigate the differential activation levels of microglial subtypes in the frontal cortex of the human brain in the AD pathology and control groups and assessed the associations of microglial subtype activation with brain pH. We hypothesized that the pH value of AD brain tissue changes, which may be related to related pathological changes and the degree of neuroinflammation in the brain.

## Results

### Decreased pH value in postmortem brain tissues from aging donors and patients with AD pathology

**Fig. 1:**
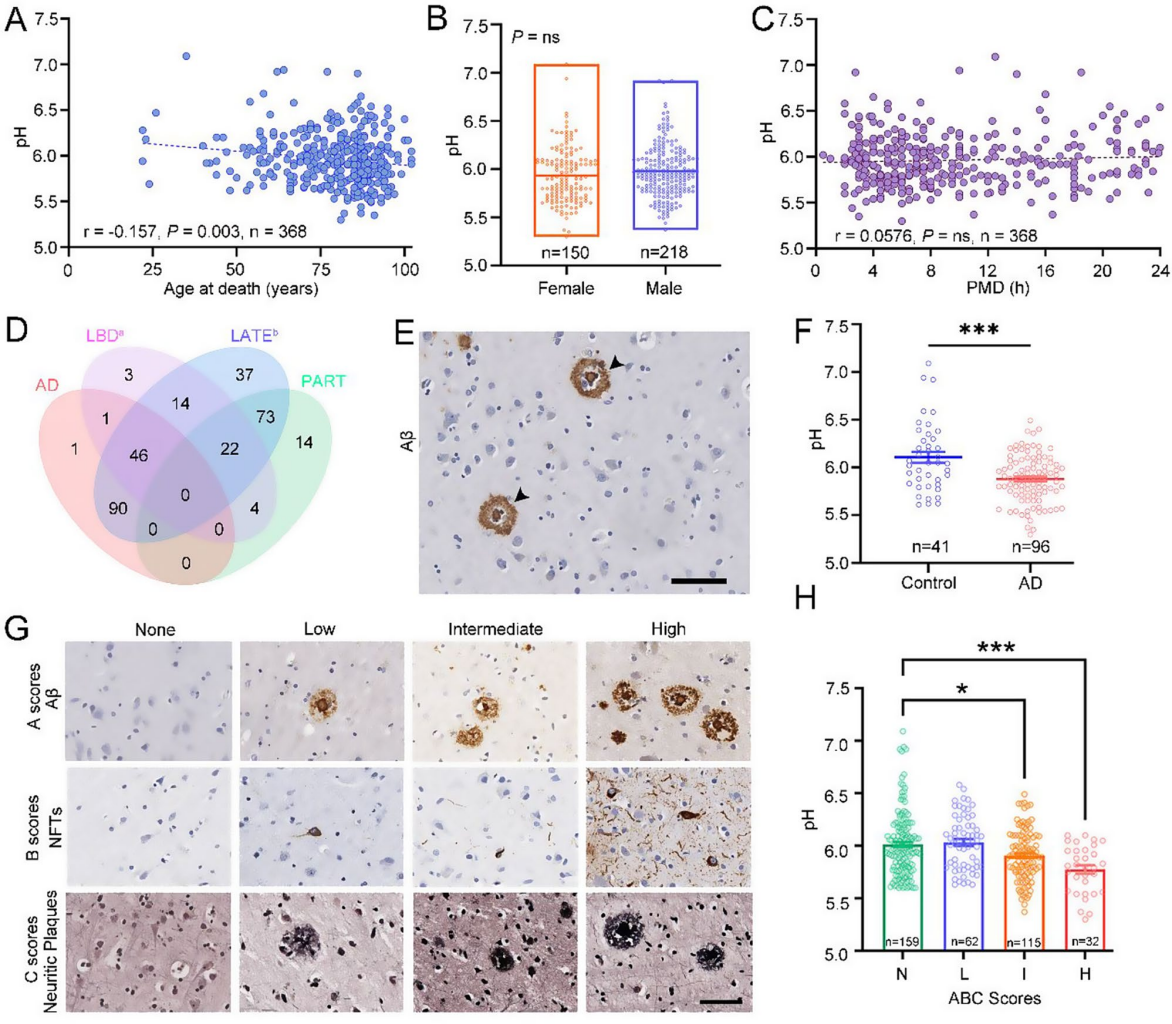
Correlations of demographic variables and neurodegenerative diseases with the pH of human postmortem brain tissue. (**A**) Correlation between the brain pH value and age at death. (**B**) Mann‒Whitney test between pH and sex. (**C**) Correlation between pH and PMD. (**D**) Overlap pathology in the AD, LBD, LATE, and PART groups. a, Twelve data points missing for the LBD group out of the 368 samples. b, Twelve data points missing for the LATE group out of the 368 samples. (**E**) Frontal lobe tissue from AD patients exhibiting Aβ expression; scale bar = 50 μm. (**F**) Mann‒Whitney test between pH and AD pathology. (**G**) Immunohistochemical staining of AD pathological markers revealed the A, B, and C scores, as well as the AD pathological grading, of the frontal lobe; scale bar = 50 μm. (**H**) Kruskal‒Wallis test between ABC scores and pH values. N = None, L = Low, I = Intermediate, H = High

The correlation between the pH value of human postmortem brain tissues from 368 donors and age at death was determined via Pearson correlation analysis. The brain pH gradually decreased with increasing age (Pearson *r* = -0.157, *P* = 0.003) (Fig. 1A). The Mann‒Whitney test revealed no difference in brain pH according to sex (Fig. 1B). Postmortem delay (PMD) of brain tissue does not significantly influence brain pH through correlation analysis (Fig. 1C). Causes of death were classified into seven categories according to the donor’s medical history, yet no significant difference in brain pH was observed (Fig. S1A and Table 1).

**Table 1:** Demographic and post-mortem characteristics of control and disease groups

Group	Case	Gender Famale: Male	Age Mean ± SD	PMD Mean ± SD	Storage time (month) Mean ± SD	pH Mean ± SD
Control	41	17:24	56.02 ± 16.42	9.79 ± 6.32	60.59 ± 38.45	6.11 ± 0.37
AD	96	55:41	85.29 ± 6.73	12.85 ±13.48	44.45 ± 31.54	5.88 ± 0.25
LBD^a^	44	15:29	83.57 ± 11.12	13.89 ± 13.18	50.45 ± 32.49	6.01 ± 0.26
LATE^b^	110	33:77	78.98 ± 10.49	11.33 ± 11.22	47.07 ± 29.17	6.00 ± 0.27
PART	89	25:64	78.25 ± 11.46	11.18 ± 10.76	49.07 ± 31.93	6.00 ± 0.29

Abbreviations: PMD, post-mortem delay; AD, Alzheimer’s disease brain pathology; LBD, Lewy body disease brain pathology; LATE, Limbic predominant age-related TDP-43 encephalopathy; PART, Primary age-related tauopathy

a, 12 data missing for LBD group out of the 368 samples

b, 12 data missing for LATE group out of the 368 samples

Regarding the proportion of comorbidities in the 368 brain samples (Fig. 1D and Table S1), we observed changes in AD pathology in 96 samples (Fig. 1E and G) and changes in LBD pathology in 44 samples. Importantly, the presence of AD brain pathology changes precluded the coexistence of LBD brain pathology changes (Fig. S1B-C). Compared with the normal control group, the Mann‒Whitney test revealed that the AD group presented a significant decrease (U = 1268, *P* = 0.0009) (Fig. 1F), but the LBD group presented no difference in brain pH (Fig. S1D). LATE pathology changed in 110 samples (Fig. S1E-F), and PART pathology changed in 89 samples (Fig. S1G-H). Mann‒Whitney tests were conducted to examine the correlations between pH and LATE or PART pathology in neurodegenerative diseases. A downward trend but no significant difference in brain pH was found between the two diseases (Fig. S1F and H).

Further analysis was conducted to determine differences in brain pH, age at death, and sex according to the type of neurodegenerative disease. A slight decrease in brain pH was detected only in the male AD group compared with the male control group (Fig. S2A-D). Although a significant negative correlation between age at death and brain pH was observed in 368 brain samples (Fig. 1A), this relationship was not evident when the data were sorted by disease group, suggesting that brain pH is not significantly influenced by aging in each disease category (Fig. S2E-I). Furthermore, compared with the control group, the disease groups were significantly older at death (Fig. S2J).

### Brain pH is strongly correlated with the degree of AD pathology

The relationships among pH, age at death, and various disease models were investigated via multiple linear regression. Table 2 presents the analysis results, which, consistent with Fig. 1A, reveal a negative correlation between age and pH in Model 1. However, when AD was included as an additional independent variable in Model 2, a stronger correlation between AD and pH was observed. This pattern was not observed for the other diseases: LBD (Model 3), LATE (Model 4), and PART (Model 5). These findings suggest that the negative correlation between pH and age at death may be significantly influenced by the presence of disease, particularly AD.

**Table 2:** Multiple linear regression analysis between pH, age at death and disease groups

Variable	Model 1	Model 2	Model 3	Model 4	Model 5
Age at death	-0.003** (0.001)	-0.002* (0.001)	-0.003** (0.001)	-0.003** (0.001)	-0.003** (0.001)
AD		-0.095** (0.034)			
LBD^a^			0.073 (0.045)		
LATE^b^				0.052 (0.032)	
PART					0.053 (0.034)
n	368	368	356	356	368
adj.R-sq	0.025	0.045	0.033	0.029	0.031
AIC	99.53	93.64	102.3	104.7	99.03
BIC	107.4	105.4	114	116.3	110.8
P value	0.002	< 0.001	0.003	0.005	0.003

Model 1 includes pH and age at death; Model 2 includes pH, age at death and AD; Model 3 includes pH, age at death and LBD; Model 4 includes pH, age at death and LATE; Model 5 includes pH, age at death and PART; Coefficients represent the estimated change in the dependent variable for a one-unit change in the predictor, holding all other predictors constant. Standard Errors are in parentheses. * *P*<0.05, ** *P*<0.01, *** *P*<0.001

Abbreviations: AD, Alzheimer’s disease brain pathology; LBD, Lewy body disease brain pathology; LATE, Limbic predominant age-related TDP-43 encephalopathy; PART, Primary age-related tauopathy, AIC, Akaike information criterion; BIC, Bayesian Information Criterion

a, 12 data missing for LBD group out of the 368 samples

b, 12 data missing for LATE group out of the 368 samples

AD neuropathological changes were assessed according to the National Institute for Aging-Alzheimer’s Association (NIA-AA) guidelines, with the “ABC” score shown in Fig. 1G. The Aβ deposition stage, according to the Thal phase scheme, is represented by A scores. A scoring system gradually aggravated the distribution of Aβ deposition from the neocortex to the entorhinal region, from the hippocampus to the basal ganglia, and finally to the midbrain and cerebellum in the brain. B scores, which are determined via the Braak criteria, reveal neurofibrillary changes in the brain, which first invade the transentorhinal and entorhinal regions and gradually accumulate in the neocortex, finally reaching the secondary and primary neocortex. The scoring of neuritic plaques, according to the CERAD scheme, is represented by C scores, which represent the CERAD plaque densities from sparse to moderate and finally frequent.

Brain pH is correlated with the pathological severity of AD. Compared with that in the no-change group (N), brain pH was slightly lower in the intermediate changes in the AD group and significantly lower in the high-change group (Kruskal‒Wallis statistic = 24.09, *P* < 0.001) (Fig. 1H). Correlations between each AD brain pathology score and pH value were analyzed via the Kruskal‒Wallis test. A significant decrease in brain pH was observed with more severe Aβ deposits in the A3 group than in the A0 group (Kruskal‒Wallis statistic = 16.14, *P* < 0.001) (Fig. S3A). A mildly significant difference was detected between B3 and B0 changes in B scores (Kruskal‒Wallis statistic = 20.93, *P* = 0.016) (Fig. S3B). A slight decrease in C scores was also observed between the C2 and C0 groups (Kruskal‒Wallis statistic = 9.874, *P* = 0.020) (Fig. S3C).

### Brain pH differs for the cognitive status of AD pathology

**Fig. 2:**
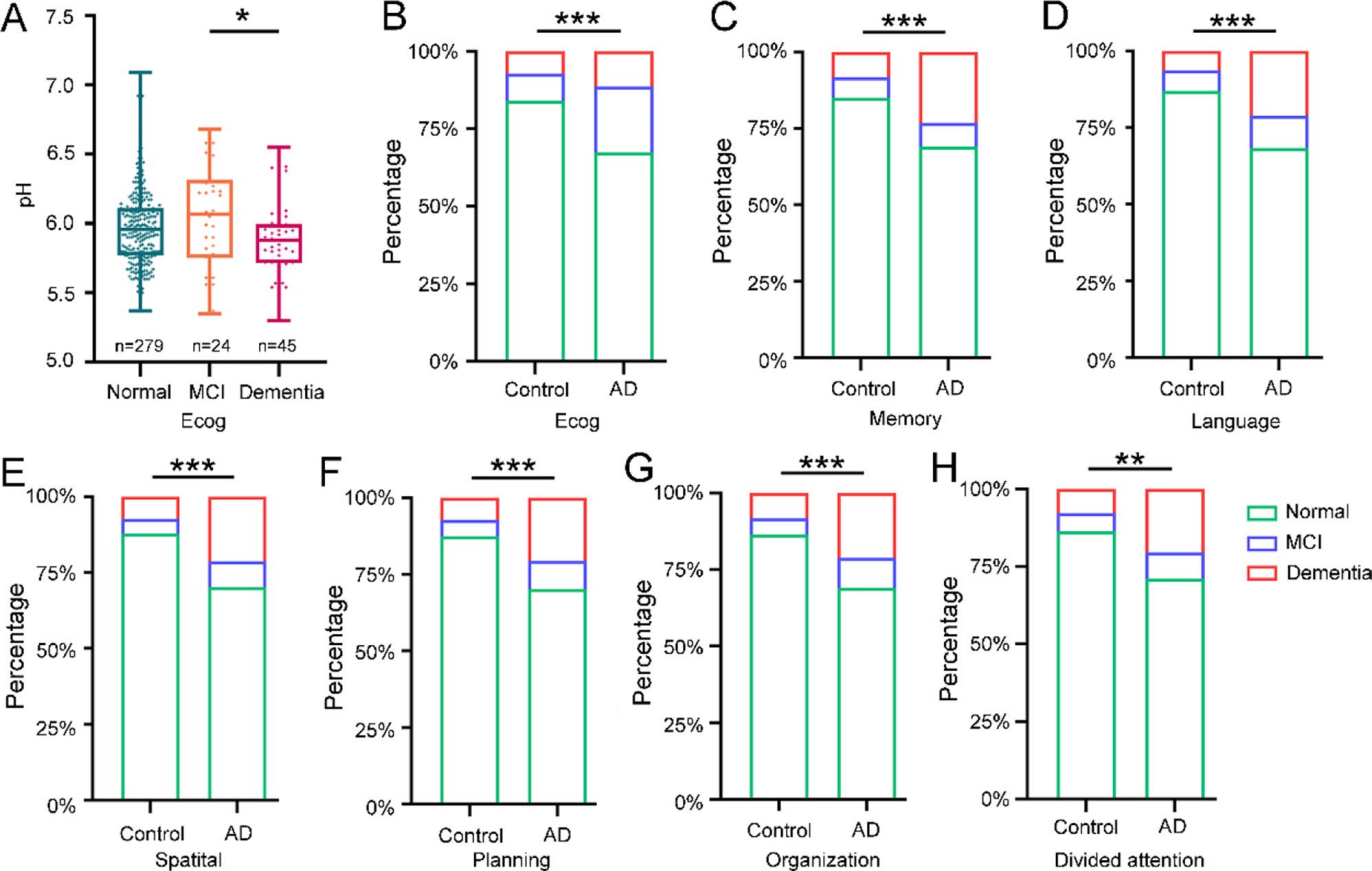
Kruskal‒Wallis test between pH and cognitive status and contingency of cognitive status in the AD and control groups. (**A**) Kruskal‒Wallis test between pH values and Ecog. (**B**) Percentage of Ecog. (**C**) Percentage of memory. (**D**) Percentage of language. (**E**) Percentage of spatial. (**F**) Percentage of planning; (**G**) percentage of organization. (**H**) Percentage of divided attention

Differences in brain pH have also been found with respect to cognitive status. Cognitive status (Ecog) data consisting of six domains were collected through questionnaire interviews with donor relatives. The Kruskal‒Wallis test was used to evaluate the difference in brain pH among the three kinds of cognitive states (Normal, MCI, and Dementia), and the Chi‒square test was used to analyze the difference in brain pH between the control and AD groups. A slight difference in brain pH was found between donors with dementia and those with MCI, and a mildly significant difference in the Ecog score was detected in the pH between the donors with dementia and those with MCI (Kruskal‒Wallis statistic = 6.485, *P* = 0.039) (Fig. 2A). Specifically, only the Language domain showed a slight difference in brain pH among the six Ecog domains between the normal and dementia groups and between the MCI and dementia groups (Kruskal‒Wallis statistic = 7.461, *P* = 0.024) (Fig. S3D-I), which differed from the results of the Ecog analysis for the control and AD groups.

Conversely, when the contingency of cognitive status in the control and AD groups was examined, a significant difference in the overall Ecog score was observed (*P* = 0.0008, chi-square = 14.23) (Fig. 2B). Additionally, all six domains of the Ecog differed: Memory, *P* = 0.0003, Chi-square = 16.14 (Fig. 2C); Language, *P* < 0.0001, Chi-square = 20.22 (Fig. 2D); Spatial, *P* = 0.0001, Chi-square = 17.64 (Fig. 2E); Planning, *P* = 0.0002, Chi-square = 16.72 (Fig. 2F); Organization, *P* = 0.0003, Chi-square = 16.09 (Fig. 2G); and Divided attention, *P* = 0.0011, Chi-square = 13.70 (Fig. 2H). These results suggest that there are distinct patterns of associations between cognitive state and brain pH and AD pathology, as well as between specific cognitive domains and pH value and AD pathology.

### The number of microglial cell subtypes differed with brain pH in the AD group

**Fig. 3:**
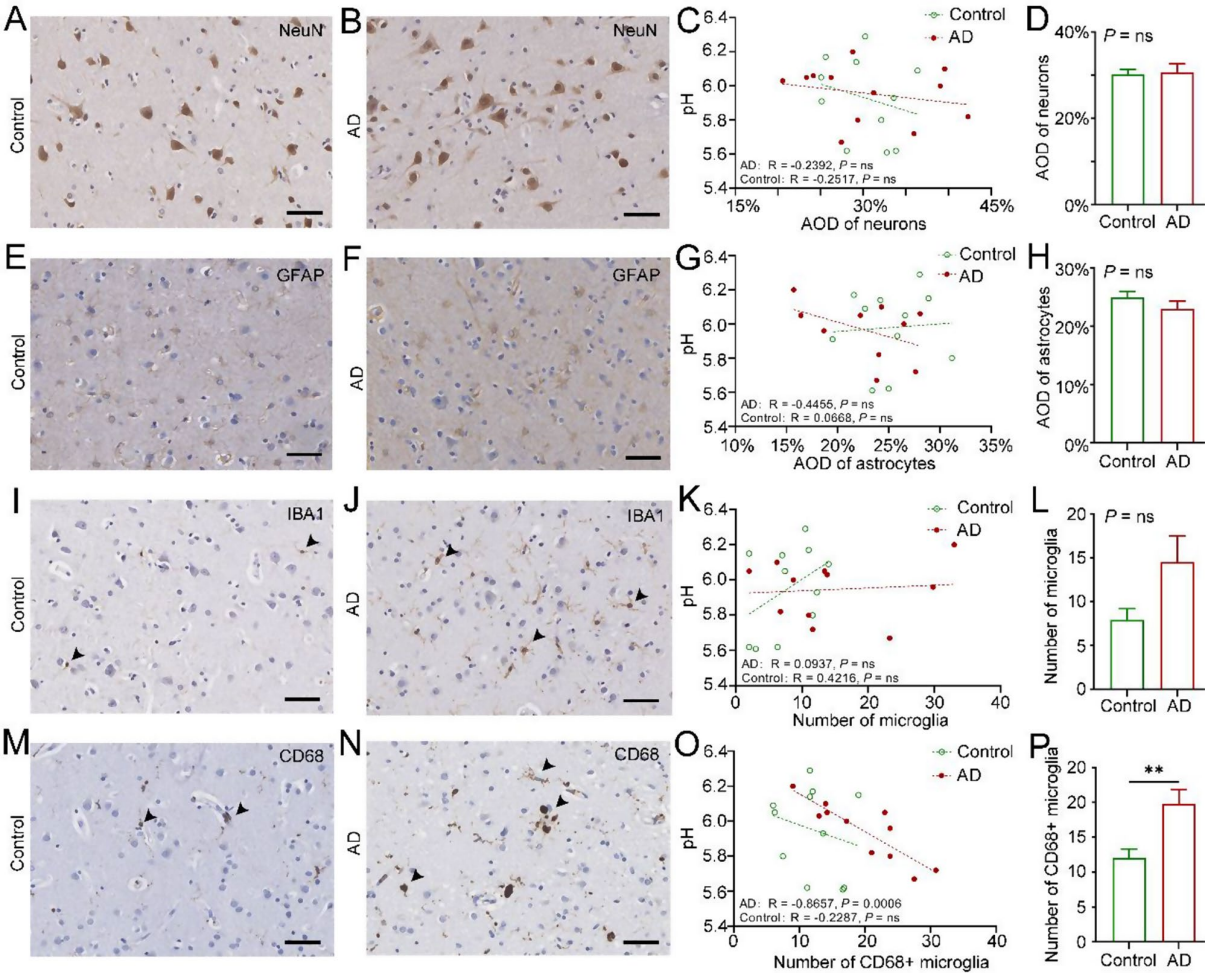
Correlations between microglial cell subtypes, neurons, astrocytes, brain pH values, and AD neuropathology. (**A**-**B**) Frontal lobe tissue from the control and AD groups exhibiting NeuN expression. (**C**) Correlations between brain pH and the AOD of NeuN in the control and AD groups. (**D**) Differences in the AOD of NeuN between the control and AD groups. (**E**-**F**) Frontal lobe tissue from the control and AD groups exhibited GFAP expression. (**G**) Correlations between brain pH and the AOD of GFAP in the control and AD groups. (**H**) Differences in the AOD of GFAP between the control and AD groups. (**I**-**J**) IBA1 expression in frontal lobe tissue from the control and AD groups. (**K**) Correlations between brain pH and the number of IBA1^+^ microglia in the control and AD groups. (**L**) Differences in the number of IBA1^+^ microglia between the control and AD groups. (**M**-**N**) CD68 expression in frontal lobe tissue from the control and AD groups. (**O**) Correlations between brain pH and the number of CD68-positive microglia in the control and AD groups. (**P**) Differences in the number of CD68-positive microglia between the control and AD groups. Scale bar = 50 μm

Given that the above results did not reveal factors highly associated with pH and AD pathology, we speculated that the activation of glial cells might influence the pH of the brain. The average optical density (AOD) of neurons and astrocytes and the number of different subtypes of microglia were assessed according to the immunohistochemical results. Neither the AOD of neurons nor the AOD of astrocytes in the frontal lobe of the brain were related to pH in our study (Fig. 3A and H).

On the basis of the immunohistochemical results of IBA1 (Fig. 3I and J) and analysis of the frontal cortex of the brain, a slight increase in the number of IBA1^+^ microglia in the AD group was observed despite the correlation being too weak to reach statistical significance (t = 2.013, *P* = 0.058) (Fig. 3K-L). To investigate the potential role of microglia in brain pH decreases, further analyses were conducted using additional microglia markers, including MHCII (Fig. S4A-D), CD163 (Fig. S4E-H), and CD68 (Fig. 3M-P) in the control and AD groups. Brain pH was not affected by the number of CD163^+^ microglia or the number of MHCII^+^ microglia according to the immunohistochemical results in the frontal lobe. In contrast to the previous markers, CD68-labeled microglia were notably increased only in the AD group and demonstrated a significant negative correlation with increasing brain pH (F = 26.93, *P* < 0.001) (Fig. 3O). Furthermore, significant differences in the number of CD68-positive microglia were observed between the control and AD groups (t = 3.198, *P* = 0.005) (Fig. 3P). These results suggest that the activation of CD68-positive microglia may be associated with a decrease in brain pH.

## Discussion

Our study revealed that pH was significantly lower in human AD brain tissue than in control brain tissue. This decrease was not observed in other neurodegenerative diseases, such as LBD, LATE, or PART. Notably, brain pH was strongly correlated with Aβ pathology (A scores). To investigate the factors influencing pH in the AD brain, the number of microglia was counted, and the activation of lysosomal-related CD68 microglia increased significantly. Furthermore, the increase in CD68 activation is associated with a decrease in brain pH. As a result, the increase in lysosomal CD68^+^ microglia may play a role in the progression of AD pathology.

The decrease in pH within the AD brain is a phenomenon that is consistent with findings from other studies in the UK, America and the Netherlands despite potential influences from genetic, environmental, and lifestyle factors [16, 27]. The consistency across different populations suggests a robust association between AD and brain pH reduction, and this decrease in pH occurred gradually with age in this study, a phenomenon that has also been confirmed in the brains of several-month-old mice [12, 16, 28]. One possible explanation is that AD may disrupt the normal age-related pH trajectory, possibly through disease-specific pathophysiological mechanisms. Despite the older age of the AD population [29], potentially introducing confounding factors, the disease’s significant impact on lowering pH levels is clear.

Although reports on the role of pH in AD progression are still emerging, evidence is mounting that supports a concern. Notably, studies have shown that infusing the mouse brain with artificial CSF titrated with HCl leads to a significant increase in Aβ deposition [16]. Additionally, pH was negatively correlated with the Braak stage in Hagihara’s study [27], suggesting that a decrease in pH is associated with AD pathology. In our present study, we identified a notable correlation between low pH values and severe Aβ pathology in human brain tissue. Additionally, lower pH values are also linked to more severe phosphorylated Tau neurofibrillary tangles and neuritic plaques, suggesting that the decreased pH value in the AD brain may be a consequence of the gradual increase in AD pathology. One possible way to decrease the pH value in the AD pathological brain, as we presented here, may be through the activation of microglia. Recent studies have demonstrated significant changes in the microglial phenotype in AD brain tissue or AD patients compared with controls [30, 31]. Variants with high expression of microglial transcripts have been identified as risk factors for AD [32, 33]. Specifically, single-nucleus RNA sequencing analysis revealed a greater-than-expected number of inflammation-related microglia in the AD brain [30]. Previous studies have shown that the number of proinflammatory microglia is increased in AD brain tissue, thereby accelerating AD pathology [32, 34–36].

The protein expression levels of IBA1, CD163, MHCII and CD68 in microglia, which represent different functional phenotypes [37, 38], were assessed in a cohort of patients with AD pathology and control donors. IBA1 is expressed in all microglia and is increased in activated microglia [39, 40]. Previous studies have shown a negative association between IBA1 microglia and dementia in the human brain, as microglia may lose the motility necessary to support neurons [41]. However, our study did not find a significant quantity variance in IBA1 microglia, which could be due to heterogeneity in AD progression and the possibility of capturing different stages of AD. CD163 represents an M2 microglia/macrophage marker that can be induced by the anti-inflammatory cytokine IL-10 [42]. There were no significant differences in the number of CD163-positive microglia between the control and AD groups in our study. However, evidence has shown that CD163 expression significantly increases in the early weeks following a lesion, both in the core and the surrounding area [43]. This finding indicates that CD163 expression patterns in AD may be more complex and not solely indicative of changes in brain pH. MHCII (HLA-DR) is expressed on the surface of antigen-presenting cells. MHCII-immunoreactive microglia/macrophages are a classical activation phenotype of microglia/macrophages [39]. CD68 is one of the most useful markers for actively phagocytic microglia/macrophages [39, 40]. A greater density of activated microglia (MHCII and CD68) was observed in AD patients with decreasing pH, particularly CD68^+^ microglia, which were significantly different between the control and AD groups. This finding is consistent with previous studies [41, 44], indicating that CD68^+^ microglia, which are predominantly expressed in lysosomes, play a role in clearing damaged cellular material and are positively associated with AD pathology.

We noted increased CD68 expression in microglia, which was correlated with inflammation and changes in brain pH. Transcriptomic data highlight the heterogeneity of endolysosomal phenotypes within microglia in both aged control and AD brains, suggesting a link between lysosomal dysfunction and aging, as well as age-related neurodegenerative diseases [30, 45–47]. Additionally, transcriptomic analysis revealed the upregulation of lysosomal pathway genes in the microglia of Tau transgenic mice [47, 48]. Lysosomes are intracellular organelles responsible for degrading protein aggregates and other large molecules in cells with pH-regulating functions [47]. Recent research has identified lysosomal acidification impairment as an early event in AD, preceding neurodegeneration and advanced pathological changes [49, 50]. This impairment affects microglia, which rely on optimal lysosomal acidification to perform their phagocytic and autophagic clearance of cellular waste and toxic proteins [11, 51]. It is essential to explore how lysosomal acidification impairment might affect the overactivation of CD68^+^ microglia and brain pH. Further research is needed to elucidate the complex interactions between microglial activation, lysosomal function, and disease progression in AD to clarify whether lysosomal acidification impairment conflicts with decreased pH values in AD brains.

Neuronal hyperexcitation was observed in different transgenic Aβ-amyloidosis mouse models before Aβ deposition and hypoactivation at later stages [52–54]. Elevated levels of lactate, a byproduct of glycolysis, serve as a marker for metabolic shifts during neuronal excitation and can lead to reduced brain pH, a factor associated with several neuropsychiatric conditions [55]. Neuronal hyperexcitation is associated with increased lactate levels in the early stage of AD, which may contribute to the observed decrease in brain pH. Previous studies have shown that overexcited neurons can release proinflammatory factors and induce microglial activation [54], potentially leading to neurotoxicity and accompanying a reduction in brain pH. Although our study did not detect any differences in the expression of neuronal (NeuN) and astrocyte (GFAP) markers related to pH changes or AD pathology, there is evidence linking parvalbumin (PV) interneurons to the pathogenesis of AD [56, 57]. PV interneurons are inhibitory interneurons that precisely control local circuitry, brain networks and memory processing [56]. In part, PV interneuron loss and impairment of AD pathogenesis are mediated by activated microglia [56, 58, 59]. Evidence indicates that mice with disruption of the Na+/H + exchanger Nhe1 in parvalbumin neurons display epileptic activity [60], which implies that parvalbumin neurons are pH dependent; further exploration is needed regarding the changes in parvalbumin neurons during the progression of AD, especially their relationship with brain pH.

Our findings indicate that CD68, a subtype of microglia related to lysosomal function, is correlated with decreased pH in the AD group. The activation of CD68 in microglia can lead to an excessive immune response, causing chronic inflammation in the brain. This process may disrupt brain tissue homeostasis and potentially affect the pH of brain tissue.

## Materials and methods

### Human brain resources

Postmortem brain tissue samples from a total of 368 donors were obtained from the National Human Brain Bank for Development and Function, Institute of Basic Medical Sciences, Chinese Academy of Medical Sciences. Detailed information, including demographic variables, ABC scores, and neuropathologic assessment information for all donors, is listed in Supplementary Table 1. The frontal pole was chosen for pH value measurement because it is known to represent the pH value of the entire brain tissue [16, 61, 62]. For immunohistochemical staining, the middle frontal gyrus was chosen for analysis. All the brain tissue sampling procedures strictly adhered to the Standardized Operational Protocol for Human Brain Banking in China [63, 64], and all the brain samples were subjected to a thorough review of the postmortem neuropathological examination results. The present study was approved by the Institutional Review Board of the Institute of Basic Medical Sciences, Chinese Academy of Medical Sciences (Approval Numbers: 009-2014 and 2022125).

### Measurement of human brain pH

A total of 368 frozen frontal cortex brain samples were included in this experiment, and each sample had an ABC pathological score to assess AD neuropathology. The brain pH was measured via a pH meter (SevenCompact™ S210, Mettler Toledo, Switzerland) after three-point calibration (pH 4.01, pH 7.00, pH 9.21). First, 100 mg of frozen brain tissue from the frontal lobe at -80 °C was placed into a 1.5 ml EP tube. The tissue was subsequently transferred to a centrifuge tube, and two grinding steel balls were added. The tissue was homogenized via a homogenizer (Homogenizer™ Bioprep-6, AllSheng, Hangzhou, China) to obtain brain homogenates. Finally, the pH value of the brain homogenate, which represents the extracellular pH value, was measured.

### Immunohistochemistry

The intensity of microglia was examined in 22 brain tissue sections covering the frontal cortex of the control (n = 11) and AD (n = 11) groups. Formalin-fixed, paraffin-embedded (FFPE) 5 µm-thick frontal sections were used for immunohistochemistry. The tissue sections were rehydrated through the addition of graded alcohol solutions and water. After antigen retrieval and saturation, the sections were blocked with a 3% hydrogen peroxide solution to reduce nonspecific staining. The sections were then incubated with the relevant primary antibody overnight at the optimal dilutions (Supplementary Table 2), followed by incubation with horseradish peroxidase (HRP)-labeled secondary antibodies (Polink-2 plus, ZSGB-BIO PV-9001 and PV-9002). A chromogenic reaction with DAB (3,3’-diaminobenzidine peroxidase substrate kit, BOSTER AR1022) was subsequently performed. Counterstaining was performed with hematoxylin to visualize the cellular and tissue structure. The dehydrated sections were cleared in xylene before being mounted with a coverslip using mounting medium (natural gum).

Neuropathology was assessed with primary antibodies directed against Aβ (β-amyloid deposition), p-tau (neurofibrillary tangle and neuritic pathology), a-synuclein (Lewy body pathology) and pTDP43 (limbic predominant age-related TDP-43 encephalopathy, LATE pathology). The activation of microglia was assessed via primary antibodies against CD68 (cluster of differentiation 68), major histocompatibility complex class II (MHC-II), cluster of differentiation 163 (CD163), and ionized calcium-binding adapter molecule 1 (IBA1). Neurons were evaluated via the neuronal marker NeuN (neuron-specific nuclear protein), whereas astrocytes were assessed via GFAP (glial fibrillary acidic protein) staining.

### Quantification

Blinded evaluation was conducted for both the control and AD groups. Analysis was conducted at 20x magnification, with three consecutive sections from each sample and 3–5 random fields of view per section, ensuring representative immunostaining detection for antibodies, including CD68, CD163, IBA1, MHCII, NeuN, and GFAP. Using ImageJ version 1.46r software, we quantified IBA1-positive microglia by counting three consecutive sections of the same brain region (frontal cortex) at 20x magnification, with 3–5 fields of view per slide, calculating the average to represent each case. This counting method was also applied to CD68, CD163, and MHCII. For GFAP and NeuN, we assessed the staining intensity using the average optical density (AOD). The images were converted to grayscale values and calibrated to the OD values in ImageJ. A consistent threshold was applied across all images from the same staining batch, including both immunized AD and nonimmunized control samples. The average density of the immunopositive area for each antibody indicated the protein load.

### Brain autopsy and neuropathologic assessments

Half of the brain tissue for neuropathologic assessment was fixed in 10% neutral buffered formalin (10% NBF) for two weeks. In accordance with the NIA-AA guidelines and SOP for Human Brain Banking in China [63, 65, 66], representative brain tissue samples were obtained for conventional paraffin embedding. The paraffin-embedded brain tissues were then sectioned at thicknesses of either 5–10 μm for subsequent immunochemical staining and special staining.

Sections were stained with hematoxylin and eosin (H&E) to facilitate diagnosis. Postmortem neuropathological assessment of AD pathology followed standardized pathological criteria [65, 66]. This involved evaluating the staging of Aβ plaque deposition on the basis of the Thal phase scheme via Aβ immunohistochemistry represented by A scores [65–67], determining the distribution of neurofibrillary tangles (NFTs) in the brain according to Braak criteria represented by B scores [68], and analyzing the frequency of neuritic plaques on the basis of pTau immunohistochemistry and special staining via the Consortium to Establish a Registry for AD (CERAD) scoring system [69]. In the LPC system, Lewy body disease (LBD) pathology is evaluated by the staging of α-synuclein-positive Lewy bodies and Lewy neurites [70]. Limbic predominant age-related TDP-43 encephalopathy neuropathologic changes (LATE) are classified by TDP-43 immunoreactivity [71, 72]. Primary age-related tauopathy (PART) brain pathology is characterized by tau biomarkers and Aβ plaques [73]. In accordance with the NIA-AA guidelines [65, 66], the overall ABC score was categorized as No (N), Low (L), Intermediate (I), or High (H). In this study, the AD group was selected on the basis of pathological assessments with I and H scores, whereas the control group was selected on the basis of N and L scores.

### Assessment of cognitive function

The Everyday Cognitive Questionnaire (ECog) is a questionnaire used to assess cognitive-related functional abilities in older adults; it consists of multiple subscales and is sensitive for detecting early functional impairment in both mild cognitive impairment (MCI) and dementia patients [74, 75]. The ECog data were collected through face‒to-face or phone interviews with the immediate kin of brain donors from all the subjects recruited from the CAMS/PUMC brain tissue bank. An average score was calculated for the overall ECog score (referred to as ECog) as well as for each separate domain. These domains include memory, language, spatial awareness, planning, organization, and divided attention [76, 77]. Cognitively normal attention was defined as an ECog ≤ 1.0. MCI was defined as an ECog ranging from 1.0 to 2.0, whereas dementia was defined as an ECog > 2.0. An ECog score of 2.0 indicates impairment in at least two cognitive domains, serving as the threshold between MCI and dementia [78].

### Statistical analysis

Statistical analyses were conducted to explore correlations involving brain pH values, demographic variables (age at death, sex), PMD, causes of death, cognitive status, and various neurodegenerative pathologies (AD, LBD, PRAT, LATE). Pearson correlation was used to examine the correlation between the brain pH value and age at death or PMD. The Mann‒Whitney test was used to assess differences in brain pH values between males and females. The Kruskal‒Wallis test was used to compare pH values among the different causes of death. The Mann‒Whitney test was used to explore the associations between pH and AD, LATE, LBD, and PART pathology. The Kruskal‒Wallis test was used to investigate pH differences across cognitive status (Normal, MCI, Dementia). The chi-square test was used to assess correlations between AD pathology and cognitive status. Simple linear regression was used to analyze the relationship between brain pH and microglial count. Student’s t test was used to compare microglial counts between the control and AD groups. Statistical analyses were performed via Stata MP (version 17.0 for Windows) and GraphPad Prism 9 (GraphPad Software, San Diego, CA). An adjusted *P value* < 0.05 indicated statistical significance (*p < 0.05, **p < 0.01, ***p < 0.001, ****p < 0.0001).

## Electronic supplementary material

Below is the link to the electronic supplementary material.

Supplementary Material 1: Table S1

Supplementary Material 2: Table S2

Supplementary Material 3: Table S3

Supplementary Material 4: Fig. S1-4

## Data Availability

All the data generated or analyzed during this study are included in this published article and its supplementary information files.

## Abbreviations

AD: Alzheimer’s disease
AOD: Average optical density
Aβ: Amyloid-beta
CD163: Cluster of differentiation 163
CD68: Cluster of differentiation 68
CERAD: Consortium to Establish a Registry for AD
CNS: Central nervous system
Ecog: Everyday cognitive questionnaire
FFPE: Formalin-fixed, paraffin-embedded
GFAP: Glial fibrillary acidic protein
H&E: Hematoxylin and eosin
HRP: Horseradish peroxidase
IBA1: Ionized calcium-binding adapter molecule 1
LATE: Limbic predominant age-related TDP-43 encephalopathy neuropathologic changes
LBD: Lewy body disease
MCI: Mild cognitive impairment
MHC: II-Major histocompatibility complex class II
NBF: Neutral buffered formalin
NeuN: Neuron-specific nuclear protein
NFTs: Neurofibrillary tangles
PART: Primary age-related tauopathy
PMD: Postmortem delay
SN: Substantia nigra
