# Supplementary material for: Changes in the pH value of the human brain in Alzheimer’s disease pathology correlated with CD68-positive microglia: a community-based autopsy study in Beijing, China
Source: Mol Brain. 2025 Feb 10;18:10. doi: 10.1186/s13041-025-01180-3 (PMC11808972; doi:10.1186/s13041-025-01180-3)
Supplement: Supplementary file 4 — Supplementary Material 4: Fig. S1-4 [file 13041_2025_1180_MOESM4_ESM.docx]

**Supplementary Figures**


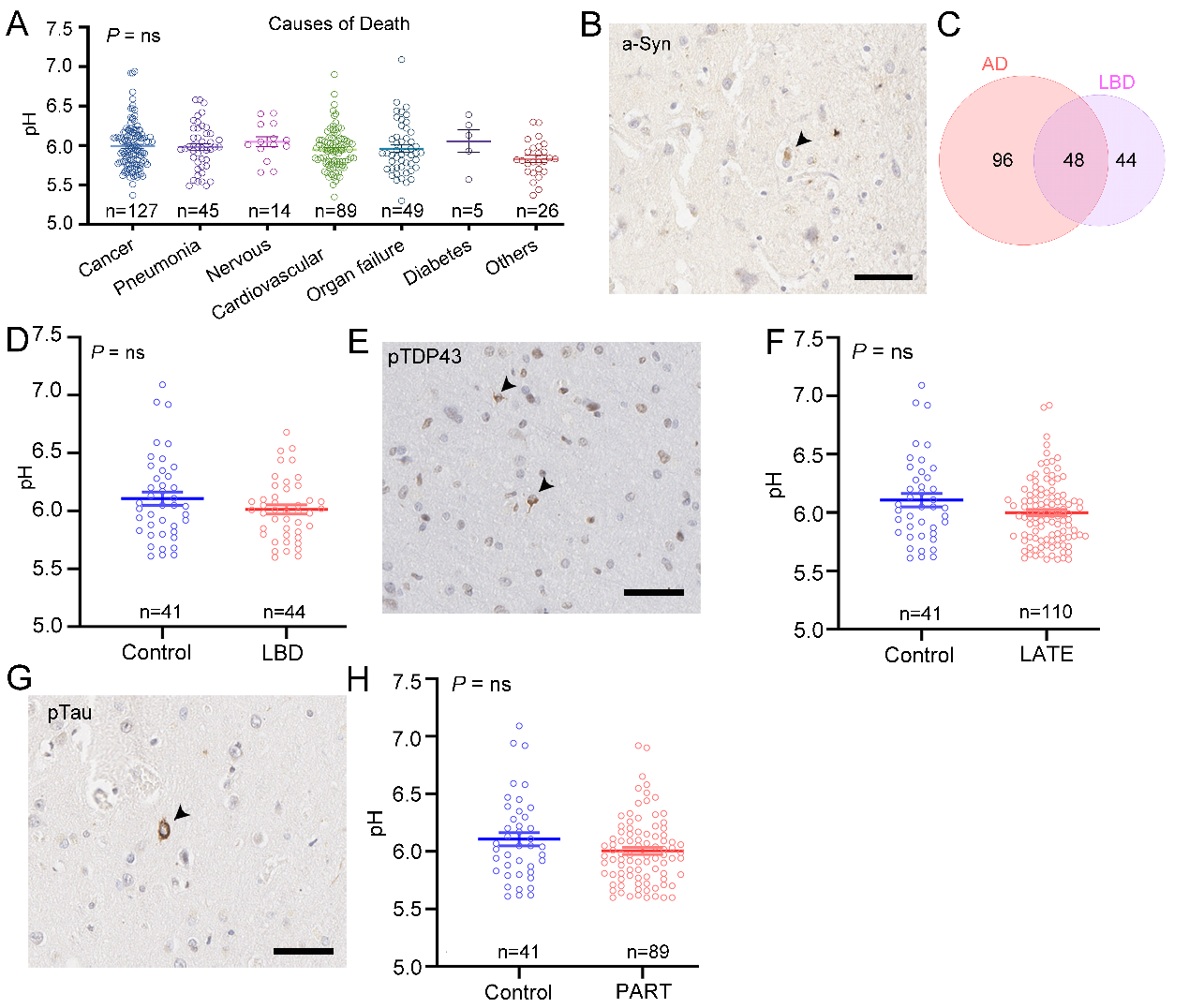


**Supplementary Figure 01. Correlations of demographic variables and neurodegenerative diseases with the pH of human postmortem brain tissue.**

**(A)** Kruskal-Wallis test between causes of death and pH values. **(B)** Frontal lobe tissue from the LBD samples exhibiting *a-*Syn expression; scale bar = 50 µm. **(C)** Overlap of brain pathology between AD patients and LBD patients. **(D)** Mann‒Whitney test between the pH value and LBD pathology. **(E)** Frontal lobe tissue from LATE samples exhibiting *p*TDP43 expression; scale bar = 50 µm. **(F)** Mann‒Whitney test between the pH value and LATE pathology. **(G)** Frontal lobe tissue from the PART samples exhibiting *p*Tau expression; scale bar = 50 µm. **(H)** Mann‒Whitney test between the pH value and PART pathology.


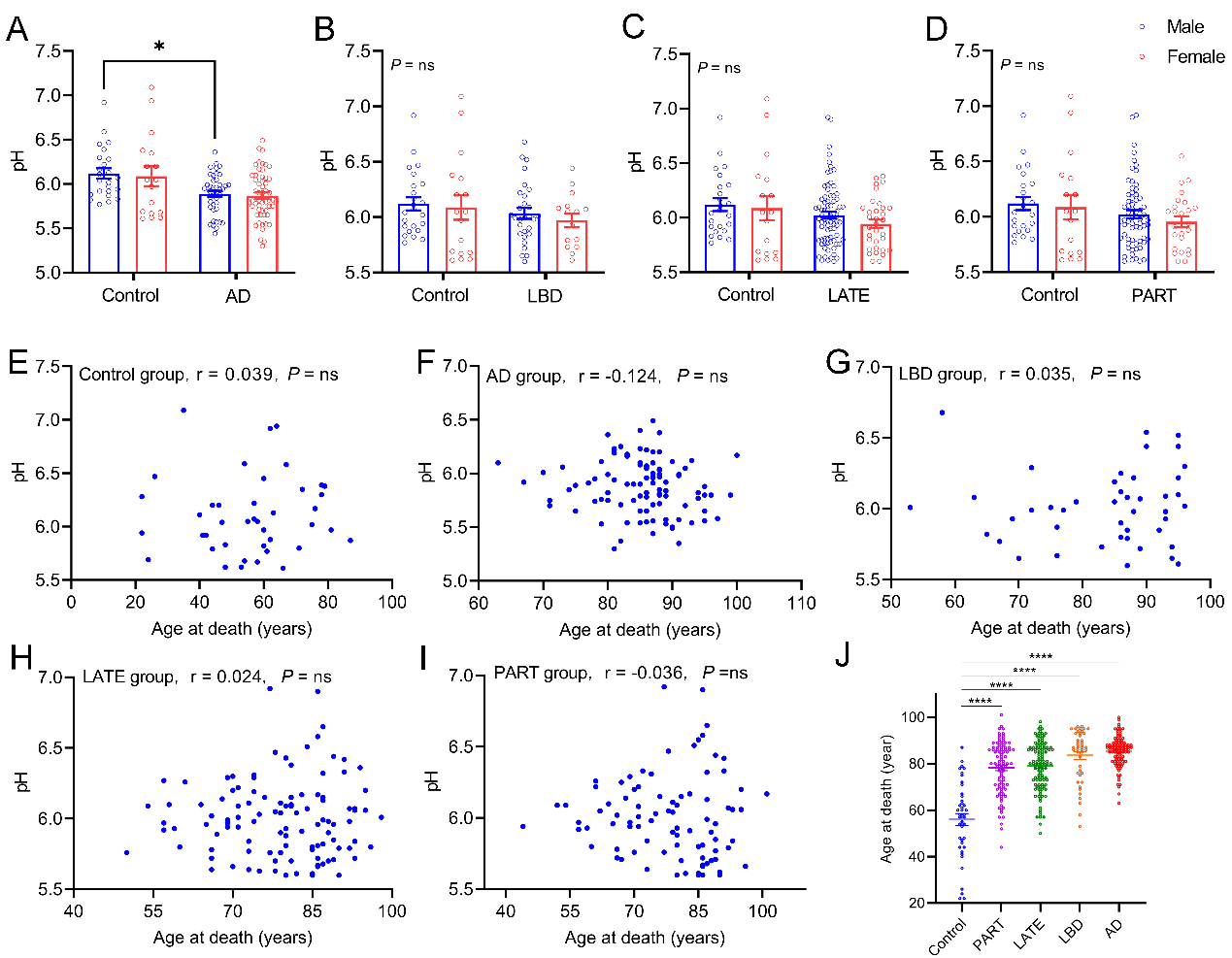


**Supplementary Figure 02. Correlations between pH and age at death.**

**(A-D)** ANOVA of pH between the disease and control groups stratified by sex subgroups for AD groups **(A)**, LBD groups **(B)**, LATE groups **(C)** and PART groups **(D)**. **(E-I)** Correlations between pH and age at death in Control group **(E)**. AD group **(F)**, LBD group **(G)**, LATE group **(H)** and PART group **(I)**.


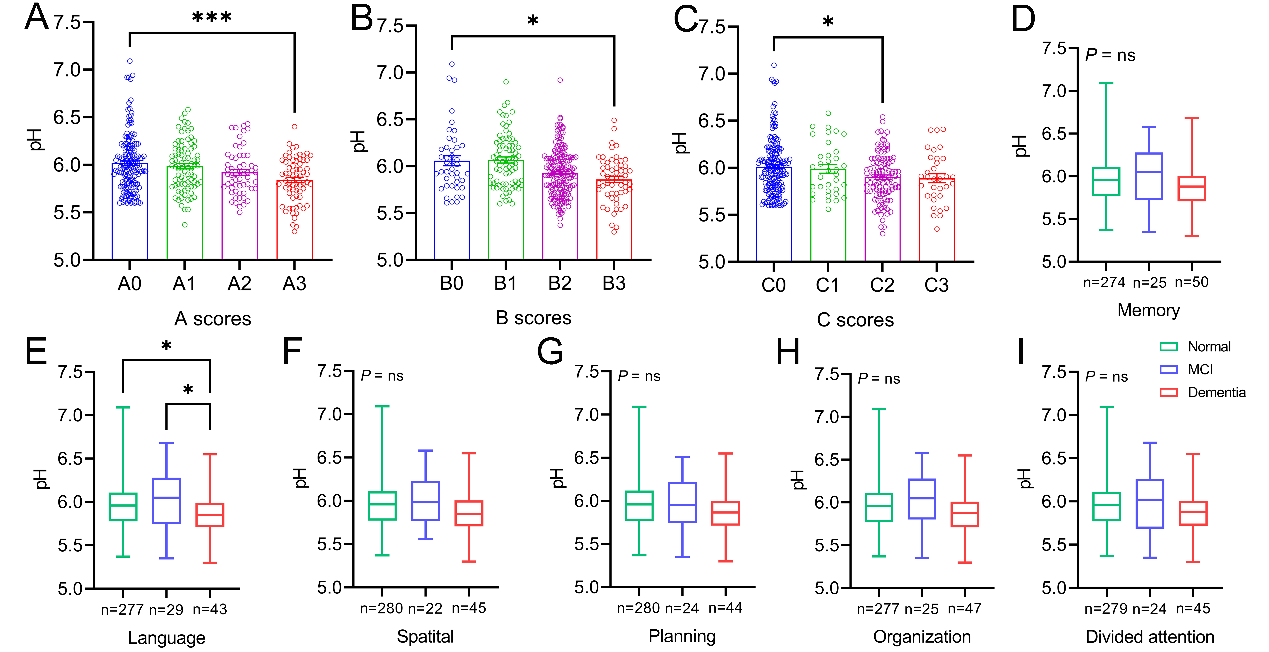


**Supplementary Figure** **03. Mann‒Whitney test between pH and AD pathology** **in the postmortem brain.**

**(A)** A scores and pH. **(B)** B scores and pH. **(C)** C scores and pH. **(D-I)** Kruskal‒Wallis test between pH and Ecog domains in the postmortem brain. pH and memory ability **(D)**, language **(E)**, spatial data **(F)**, planning **(G)**, organization **(H)**, divided attention **(I)**.

**
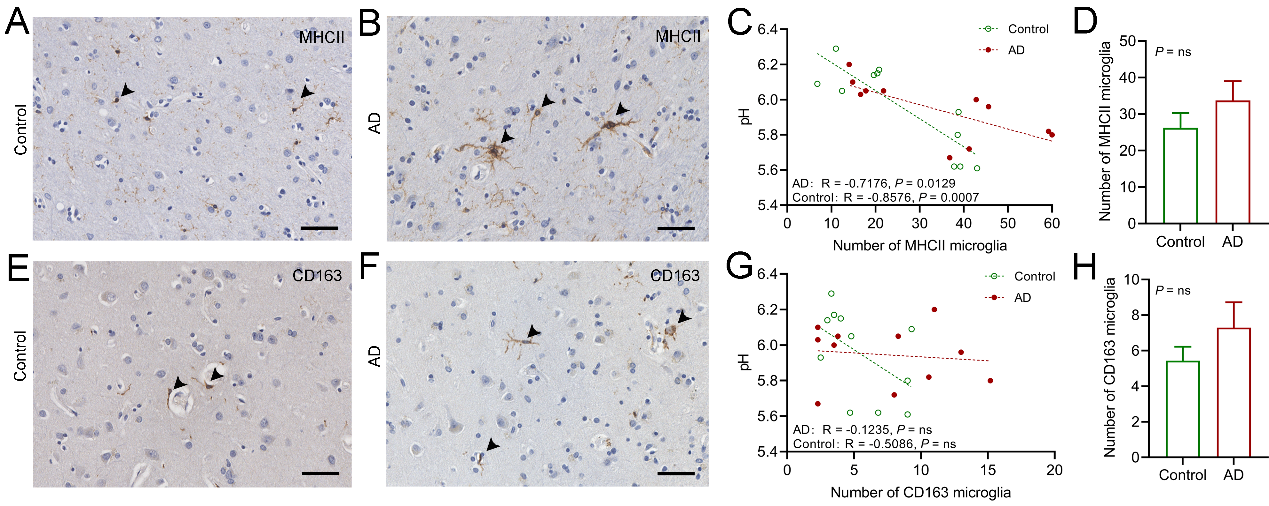
**

**Supplementary Figure 04. Correlations between the numbers of microglial cell subtypes, brain pH, and AD neuropathology.**

**(A-B)** Frontal lobe tissue from the control and AD groups exhibiting MHCII expression. **(C)** Correlations between brain pH and the number of MHCII-positive microglia in the control and AD groups. **(D)** Differences in the number of MHCII-positive microglia between the control and AD groups. **(E-F)** CD163 expression in frontal lobe tissue from the control and AD groups. **(G)** Correlations between brain pH and the number of CD163-positive microglia in the control and AD groups. **(H)** Differences in the number of CD163-positive microglia between the control and AD groups. Scale bar = 50 µm.
